# Improving the models for prognosis of aneurysmal subarachnoid hemorrhage with the neutrophil-to-albumin ratio

**DOI:** 10.3389/fneur.2023.1078926

**Published:** 2023-03-24

**Authors:** Renjie Zhang, Zheran Liu, Yu Zhang, Yiyan Pei, Yan He, Jiayi Yu, Chao You, Lu Ma, Fang Fang

**Affiliations:** ^1^Department of Neurosurgery, West China Hospital of Sichuan University, Chengdu, Sichuan, China; ^2^Department of Biotherapy, Cancer Center, West China Hospital of Sichuan University, Chengdu, Sichuan, China; ^3^Center for Evidence-Based Medical and Clinical Research, Affiliated Hospital of Chengdu University, Chengdu, Sichuan, China; ^4^School of Medical and Life Sciences, Chengdu University of Traditional Chinese Medicine, Chengdu, Sichuan, China

**Keywords:** neutrophil-to-albumin ratio, intracranial aneurysm, subarachnoid hemorrhage, mortality, prediction model

## Abstract

**Objective:**

Many peripheral inflammatory markers were reported to be associated with the prognosis of aneurysmal subarachnoid hemorrhage (aSAH). We aimed to identify the most promising inflammatory factor that can improve existing predictive models.

**Methods:**

The study was based on data from a 10 year retrospective cohort study at Sichuan University West China Hospital. We selected the well-known SAFIRE and Subarachnoid Hemorrhage International Trialists’ (SAHIT) models as the basic models. We compared the performance of the models after including the inflammatory markers and that of the original models. The developed models were internally and temporally validated.

**Results:**

A total of 3,173 patients were included in this study, divided into the derivation cohort (*n* = 2,525) and the validation cohort (*n* = 648). Most inflammatory markers could improve the SAH model for mortality prediction in patients with aSAH, and the neutrophil-to-albumin ratio (NAR) performed best among all the included inflammatory markers. By incorporating NAR, the modified SAFIRE and SAHIT models improved the area under the receiver operator characteristics curve (SAFIRE+NAR vs. SAFIRE: 0.794 vs. 0.778, *p* = 0.012; SAHIT+NAR vs. SAHIT: 0.831 vs. 0.819, *p* = 0.016) and categorical net reclassification improvement (SAFIRE+NAR: 0.0727, *p* = 0.002; SAHIT+NAR: 0.0810, *p* < 0.001).

**Conclusion:**

This study illustrated that among the inflammatory markers associated with aSAH prognosis, NAR could improve the SAFIRE and SAHIT models for 3 month mortality of aSAH.

## Introduction

Aneurysmal subarachnoid hemorrhage (aSAH) is a fatal disease (1). Between 25 and 30% of patients with aSAH die within 3 months of onset (2), and 40% of aSAH patients do not regain independent function (3). Consequently, establishing an accurate and straightforward prediction model for the early prognosis of aSAH has always been a priority in aSAH clinical research.

Two externally validated predictive models, the SAFIRE model (4) and the SAHIT model (5), have been developed using data from prospective cohort studies. The derivation cohort of the SAFIRE model included 1,215 patients, while the validation cohort included 2,143; for the SAHIT model, 10,936 and 3,355 patients were included in the respective cohorts. The area under the receiver operating characteristic curve (AUC) for SAFIRE was 0.83 (95%CI 0.80–0.85), while AUC values for SAHIT remained between 0.76–0.81 in external validation. However, *R*^2^ in SAHIT was only 23–31%, signifying that the included predictors explained only 23–31% of the variability in outcome, whereas the SAFIRE model does not report its *R*^2^. As neither model incorporated laboratory tests, the potential of baseline biomarkers to enhance the model has yet to be explored.

Recent studies have confirmed that inflammation in the initial phase of aSAH is implicated in its pathological process (6). Several peripheral inflammatory markers, including the neutrophil-to-albumin ratio (NAR) (7), neutrophil-to-lymphocyte ratio (NLR) (8), platelet-to-lymphocyte ratio (PLR) (9), monocyte-to-lymphocyte ratio (MLR) (10), and systemic immune inflammation index (SII) (11), have been reported to be associated with short-term outcomes of aSAH, raising the possibility that they may serve as promising prognosticators. However, there has yet to be a systematic study on whether these markers can improve the predictive capabilities of existing models. This study sought to investigate if these validated inflammatory markers could bolster the predictive power of SAFIRE and SAHIT models, in addition to selecting the marker that conferred the greatest enhancement.

## Methods

### Study design and source of data

Patient data were derived from a large observational cohort study at Sichuan University West China Hospital. Patients were divided into the derivation cohort (February 2009 to December 2017) and the validation cohort (January 2018 to July 2019). Treatment of patients was carried out according to standardized guidelines (12).

Patients were enrolled only when they were diagnosed with SAH by computed tomography, magnetic resonance imaging, angiography, or cerebrospinal fluid test, and aneurysm were identified precisely. Exclusion criteria included (1) aneurysms were caused by trauma or arteriovenous malformations and (2) aneurysms were treated before ictus. We also excluded patients whose personal identification numbers were wrong or whose household registrations were not found in the Household Registration Administration System. We used personal identification numbers to identify death records from this system.

The study was approved by the West China Hospital Institutional Review Board (No. 20211701), with a waiver of informed consent due to minimal risk to patients. Predictive models were reported according to the TRIPOD statement (Checklist in the Supplemental material) (11).

### Predictors

According to the SAFIRE and SAHIT models, age, medical history of hypertension, aneurysm location, aneurysm size, World Federation of Neurological Surgeons (WFNS) grade on admission, Fisher grade on admission, and methods of treatment (clip, coil, or no treatment) were collected.

Aneurysm size and Fisher grade categories were defined separately as SAFIRE or SAHIT predictive tool. In the SAFIRE model, aneurysm sizes were categorized as <10 mm, 10–19.9 mm, or ≥ 20 mm, and Fisher grades were categorized into 1–3 or 4. In the SAHIT model, aneurysm sizes were categorized as ≤12 mm, 13–24 mm, or ≥ 25 mm, and original Fisher grades were enrolled. Similarly, age was treated as a continuous variable in the SAHIT model and a categorical variable in the SAFIRE model (≤50 y, 50–60 y, 60–70 y, or ≥ 70 y). Due to the limited data, locations of aneurysms were imputed as anterior or posterior circulation.

According to the current studies, we identified five markers to predict outcomes in aSAH patients, including the NAR, NLR, PLR, MLR, and SII. Their calculation methods were presented in Supplementary Figure S1. Considering the Practical clinical application, only laboratory examination results within 24 h were selected. Multicollinearity was assessed using the variance inflation factor (VIF). A VIF value >5 indicates severe collinearity (13).

### Outcome

The outcome was defined as mortality at 3 months. All death records were extracted through the Household Registration System, which documents Chinese citizens’ death dates. The system is based on self-reporting death by relatives and the Seventh National Census, with a missing registration rate of 5 per 10,000, which was reported by the National Bureau of Statistics (14). Therefore, this system has accurate death records and bind assessments (15).

### Missing data

All data were complete except aneurysm size and Fisher grade. In the derivation cohort, missing aneurysm size and Fisher grade values were filled using multiple imputation (16) with a predictive mean matching method to generate 5 imputations. Complete case analysis was adopted in the validation cohort.

### Comparison of inflammatory markers

Considering the colinearity of the five inflammatory markers, we compared their predictive abilities before adding to the predictive models using binary logistic regression and area under the receiver operator characteristics curve (AUC). The DeLong test was employed to distinguish the difference between AUCs (17).

### Model development

Following the original SAFIRE predictive tool, binary logistic regression was adopted to establish the predictive model, and the same variables (age, aneurysm size, Fisher grade, and WFNS grade) were included. Similarly, we applied the same predictors (age, history of hypertension, aneurysm location, aneurysm size, Fisher grade, WFNS grade, and treatment) as the SAHIT model used to build a binary logistic regression model. The inflammation marker with the highest predictive value was added to these models for further development.

### Model performance

The modified models were compared with the original models. Performance between models was evaluated from different perspectives by a variety of approaches. AUC values with 95% CIs and integrated discrimination improvement (IDI) were reported to represent the discrimination significance of the modified models. Categorical net reclassification improvement (NRI) was employed to illustrate the reclassification, and decision-curve analysis (DCA) was used to show the net benefit and visualize the clinical usefulness. According to the previous study (18), we defined risk ratio < 0.1 as low, 0.1–0.6 as moderate, and > 0.6 as high risk of long-term mortality.

The *R*^2^ statistic was reported to identify the proportion of variance explained by the predictive models, and the contribution of each predictor to the predictive models was represented with the partial *R*^2^ statistic (19).

### Model calibration

The calibration plots (20) were used to evaluate the calibration of the prediction models, and the Brier score (21) was computed to measure the prediction accuracy. The Brier score ranges from 0 to 0.25. The closer the Brier score is to 0, the better the model calibration degree is. When the Brier score equals 0.25, the model has no prediction ability.

### Model validation

Two parts of model validation were completed. A 400 times 10-fold cross-validation was adopted as the internal validation strategy, and mean AUC values and average error were reported.

We compared the derivation cohort (2009–2017) and the validation cohort (2018–2019) as a temporal validation, and the AUC, IDI, and NRI values of the temporal validation cohort were calculated.

### Sample size

The sample size of this study was calculated using the formula developed by Riley et al. (22). The required minimum sample size was obtained for developing a new model using the SAFIRE predictive tool consisting of 323 patients. Using the SAHIT predictive tool, the minimum sample size was 458 patients.

### Sensitivity analysis

For further sensitivity analysis, inflammatory markers were added as categorical variables using their cut-off values, and potentially meaningful interaction effects were reported.

All analyses were conducted using R software (version 4.2.1, R Foundation for Statistical Computing). Web-based nomograms were developed using the DynNom R package.

## Results

### Patient demographics and missing data

**Figure 1:**
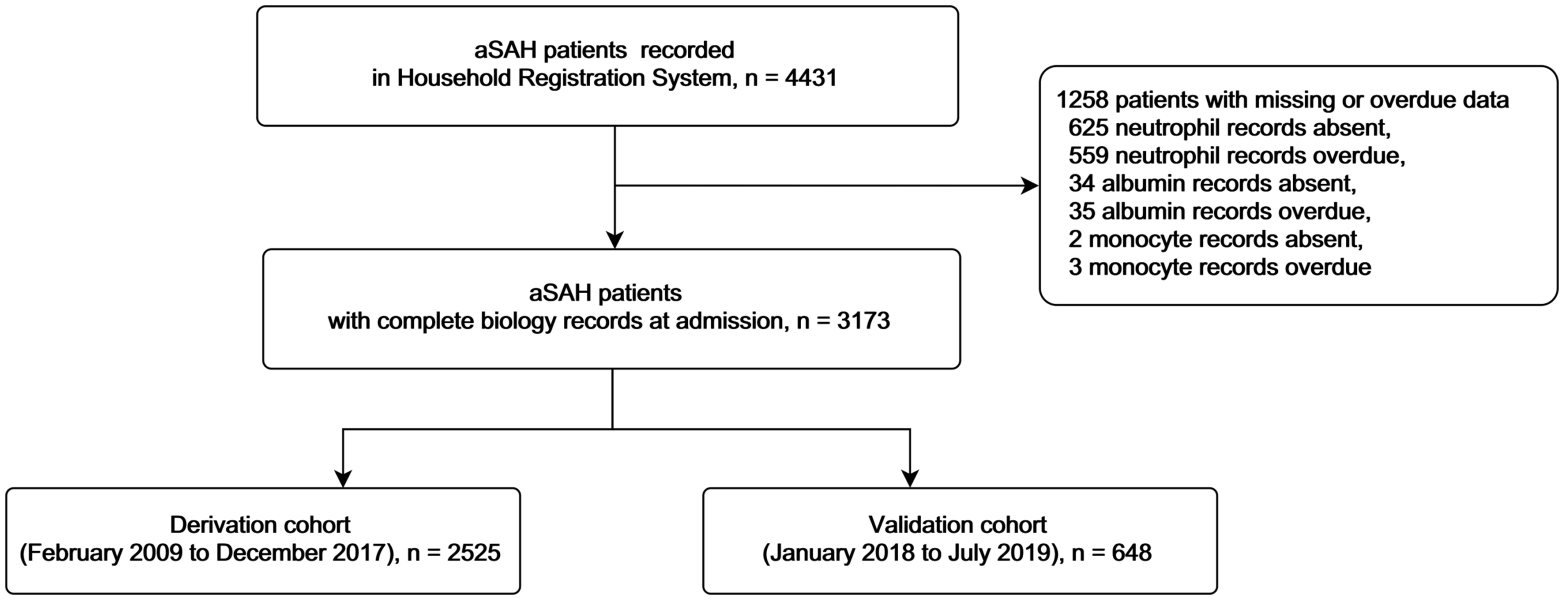
Flow chart for patients included in this study.

A total of 3,173 patients were included in this study (Figure 1 details patient inclusion flow chart). Patients were divided into the derivation cohort (*n* = 2,525) and the validation cohort (*n* = 648). The median age was 55 (interquartile range 47–63) in the derivation cohort and 55 (48–66) in the validation cohort. Most patients were female (65.3% in the derivation cohort and 63.6% in the validation cohort). Mortalities at 3 months were similar in the two cohorts (11.8% and 11.6, respectively). Details of the characteristics were summarized in Table 1.

**Table 1:** Data of patients included in the study.

Characteristics	Derivation cohort	Validation cohort	*p* value
	(*n* = 2,525)	(*n* = 648)	
*Demographics*			
Age, *n* (%)			
≤50 y	959 (38.0)	209 (32.3)	0.001
50–60 y	656 (26.0)	152 (23.5)	
60–70 y	645 (25.5)	194 (29.9)	
≥70 y	265 (10.5)	93 (14.4)	
*Medical history, n (%)*			
Hypertension	629 (24.9)	144 (22.2)	0.17
*Aneurysm characteristics*			
Posterior location, *n* (%)	360 (14.3)	241 (37.2)	<0.001
*Size of the aneurysm, n (%)*			
<10 mm	1512 (59.9)	443 (68.4)	<0.001
10–20 mm	317 (12.6)	46 (7.1)	
≥20 mm	107 (4.2)	13 (2.0)	
Missing	589 (23.3)	146 (22.5)	
*Hemorrhagic characteristics, n (%)*			
*WFNS grade*			
I	1465 (58.0)	377 (58.2)	<0.001
II	389 (15.4)	134 (20.7)	
III	75 (3.0)	4 (0.6)	
IV	290 (11.5)	35 (5.4)	
V	306 (12.1)	98 (15.1)	
*Fisher grade*			
I	116 (4.6)	22 (3.4)	0.04
II	406 (16.1)	81 (12.5)	
III	280 (11.1)	84 (13.0)	
IV	1,032 (40.9)	293 (45.2)	
Missing	691 (27.4)	168 (25.9)	
*Treatment of aneurysms, n (%)*			
Clip	1758 (69.6)	365 (56.3)	<0.001
Coil	350 (13.9)	45 (6.9)	
No treatment	417 (16.5)	238 (36.7)	
*Biology, mean (SD)*			
Neutrophil, 10^9^/L	8.78 (4.40)	9.08 (4.62)	0.12
Platelet, 10^9^/L	173.00 (70.40)	180.11 (70.80)	0.02
Lymphocyte, 10^9^/L	1.21 (0.61)	1.15 (0.58)	0.02
Albumin, g/L	39.91 (5.14)	40.36 (5.32)	0.05
Monocyte, 10^9^/L	0.53 (0.29)	0.56 (0.29)	0.06
NAR	0.22 (0.11)	0.23 (0.12)	0.28
NLR	9.79 (8.51)	10.67 (8.88)	0.02
PLR	174.41 (118.31)	193.52 (137.02)	<0.001
MLR	1.64 (1.62)	1.92 (2.06)	<0.001
SII	0.52 (0.37)	0.58 (0.42)	<0.001
*Outcome at 3 months, n (%)*			
Survivor	2226 (88.2)	573 (88.4)	0.90
Death	299 (11.8)	75 (11.6)	

NAR, neutrophil-to-albumin ratio; NLR, neutrophil-to-lymphocyte ratio; PLR, platelet-to-lymphocyte ratio; MLR, monocyte-to-lymphocyte ratio; SII, systemic immune-inflammation index.

In the derivation cohort, 23.3% of patients were missing aneurysm size values, and 27.4% were missing Fisher grade values. Any missing data in the derivation cohort were imputed. The validation cohort included 423 patients with complete data in the final computation.

### Model development

**Figure 2:**
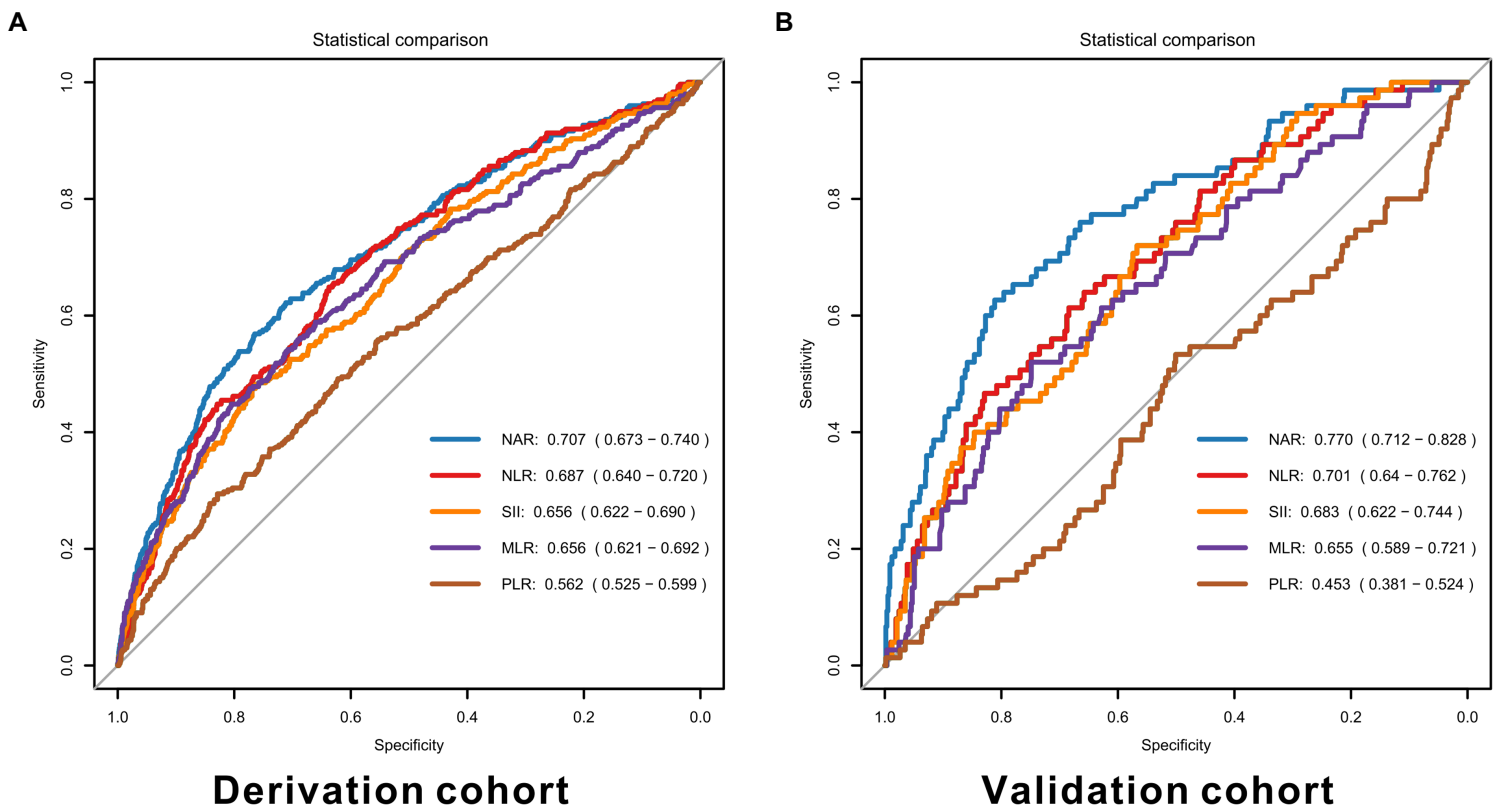
ROC curves for inflammatory markers in the derivation **(A)** and validation cohorts **(B)**. NAR, neutrophil-to-albumin ratio, NLR, neutrophil-to-lymphocyte ratio, PLR, platelet-to-lymphocyte ratio, MLR, monocyte-to-lymphocyte ratio, SII, systemic immune inflammation index.

In the derivation cohort, NAR performed the best predictive ability with the highest AUC of 0.707 (95% CI 0.673–0.740), and PLR was the weakest with the lowest AUC of 0.562 (95%CI 0.525–0.599), as shown in Figure 2. Similar results were represented in the validation cohort (NAR: AUC = 0.770, 95% CI 0.712–0.828; PLR: AUC = 0.453, 95% CI 0.381–0.524). Details of each marker’s performance were reported in Supplementary Table S1. Considering the poor performance of PLR, we did not add it to the models furtherly.

Before adding to SAFIRE and SAHIT models, the collinearity of the original model predictors and the inflammatory markers was examined *via* VIF, as shown in Supplementary Tables S2, S3. There was no VIF value over 5.

In the original SAFIRE model, the strongest predictor was WFNS grade (partial *R*^2^ = 10.02%), followed by age (partial *R*^2^ = 0.93%), Fisher grade (partial *R*^2^ = 0.73%), and aneurysm size (partial *R*^2^ = 0.40%). In the original SAHIT model, the strongest predictor was WFNS grade (partial *R*^2^ = 7.71%), followed by treatment (partial *R*^2^ = 4.35%), Fisher grade (partial *R*^2^ = 1.24%), aneurysm size (partial *R*^2^ = 1.00%), age (partial *R*^2^ = 0.23%), history of hypertension (partial *R*^2^ = 0.05%), and aneurysm location (partial *R*^2^ = 0.03%).

**Figure 3:**
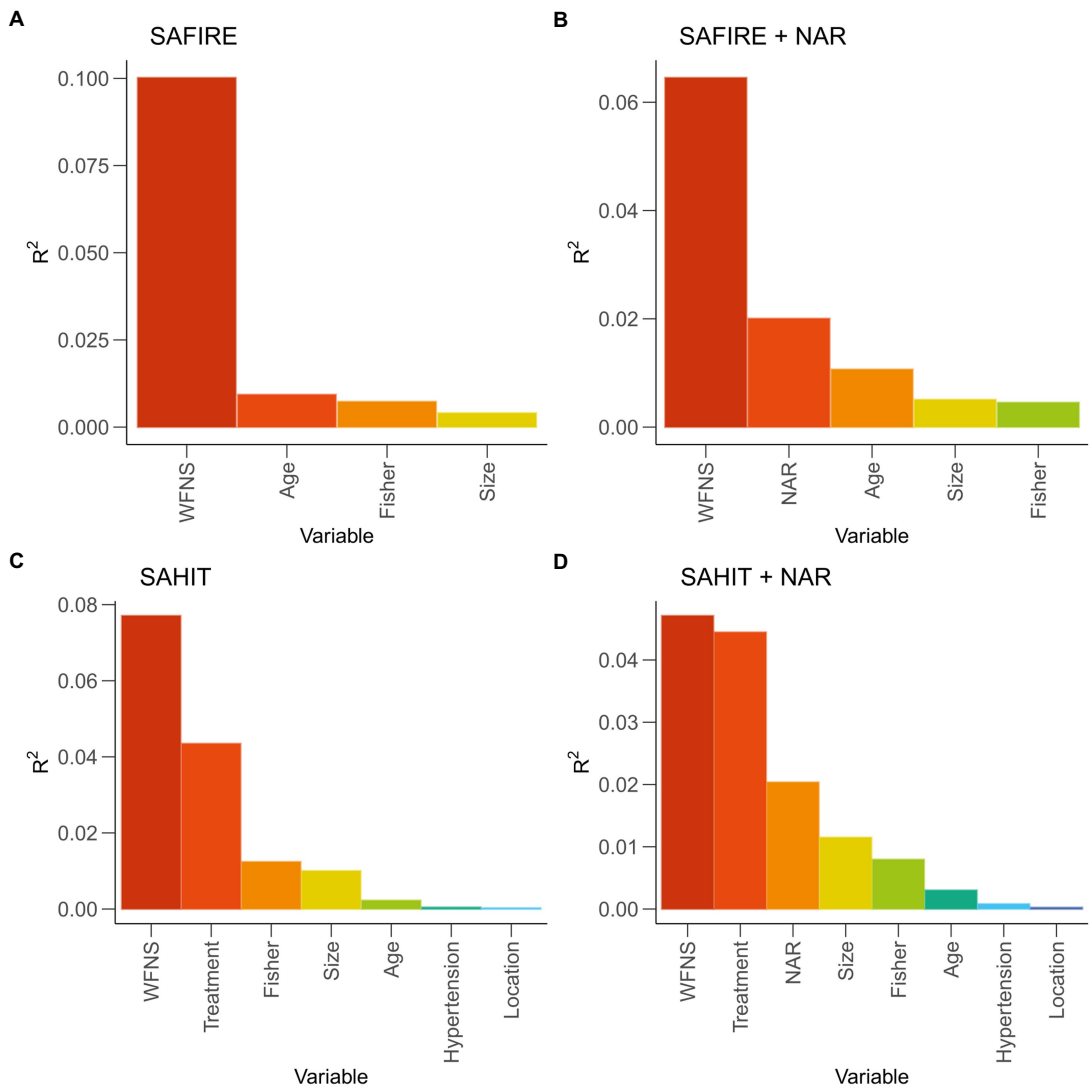
General dominance of predictors reported as partial *R*^2^ statistic in original and modified models. SAHIT, original Subarachnoid Hemorrhage International Trialists’ model; SAFIRE, the SAFIRE grading scale indicates size of the aneurysm, age, fisher grade, World Federation of Neurological Surgeons; WFNS, World Federation of Neurological Surgeons; NAR, neutrophil-to-albumin ratio.

As shown in Figure 3, NAR was the second significant predictor in the modified SAFIRE model (partial *R*^2^ = 2.01%) and the third in the modified SAHIT model (partial *R*^2^ = 2.04%). The significance of other markers after adding to the predictive models was illustrated in Supplementary Figure S2. Details of the logistic regression models were reported in Supplementary Tables S4, S5.

### Model performance

#### SAFIRE + inflammatory markers

As shown in Table 2, inputting the same predictors as the SAFIRE predictive tool generated a comparable AUC value in the derivation cohort (AUC = 0.778, 95% CI 0.750–0.906). After adding inflammatory markers, all modified predictive models achieved a better discriminative ability in the derivation cohort. Among the four markers, adding NAR to the original model acquired the highest AUC development (SAFIRE+NAR vs. SAFIRE: ∆AUC = 0.016, *p* = 0.012) and the highest IDI (SAFIRE+NAR vs. SAFIRE: IDI = 0.025, *p* < 0.001).

**Table 2:** Performance of SAFIRE and SAFIRE + inflammatory biomarkers models.

Models	Discrimination				Reclassification		Calibration
	AUC (95% CI)	*p* value	IDI (95% CI)	*p* value	Categorical NRI (95% CI)	*p* value	Brier score
*Derivation cohort*							
SAFIRE	0.778 (0.750–0.806)	Ref.	Ref.		Ref.		0.090
SAFIRE+NAR	0.794 (0.766–0.821)	0.012	0.025 (0.015–0.035)	<0.001	0.073 (0.026–0.119)	0.002	0.087
SAFIRE+MLR	0.791 (0.764–0.818)	0.012	0.014 (0.005–0.022)	<0.001	0.041 (0.006–0.077)	0.024	0.088
SAFIRE+NLR	0.790 (0.763–0.817)	0.012	0.015 (0.006–0.024)	<0.001	0.045 (0.007–0.083)	0.020	0.088
SAFIRE+SII	0.787 (0.760–0.814)	0.037	0.013 (0.006–0.020)	<0.001	0.014 (−0.019–0.047)	0.404	0.088
*Validation cohort*							
SAFIRE	0.771 (0.709–0.833)	Ref.	Ref.		Ref.		0.108
SAFIRE+NAR	0.815 (0.757–0.873)	0.001	0.057 (0.030–0.085)	<0.001	0.101 (0.003–0.199)	0.044	0.099
SAFIRE+MLR	0.787 (0.728–0.846)	0.140	0.005 (−0.012–0.022)	0.571	−0.001 (−0.070–0.069)	0.992	0.109
SAFIRE+NLR	0.782 (0.721–0.844)	0.271	0.017 (−0.001–0.035)	0.062	−0.052 (−0.130–0.025)	0.185	0.106
SAFIRE+SII	0.780 (0.718–0.841)	0.385	0.022 (0.002–0.041)	0.033	−0.014 (−0.103–0.075)	0.759	0.106

NAR, neutrophil-to-albumin ratio; MLR, monocyte-to-lymphocyte ratio; NLR, neutrophil-to-lymphocyte ratio; SII, systemic immune-inflammation index; AUC, the area under the receiver operator characteristics curve; IDI, integrated discrimination improvement; NRI, net reclassification improvement; CI, confidence interval.

**Figure 4:**
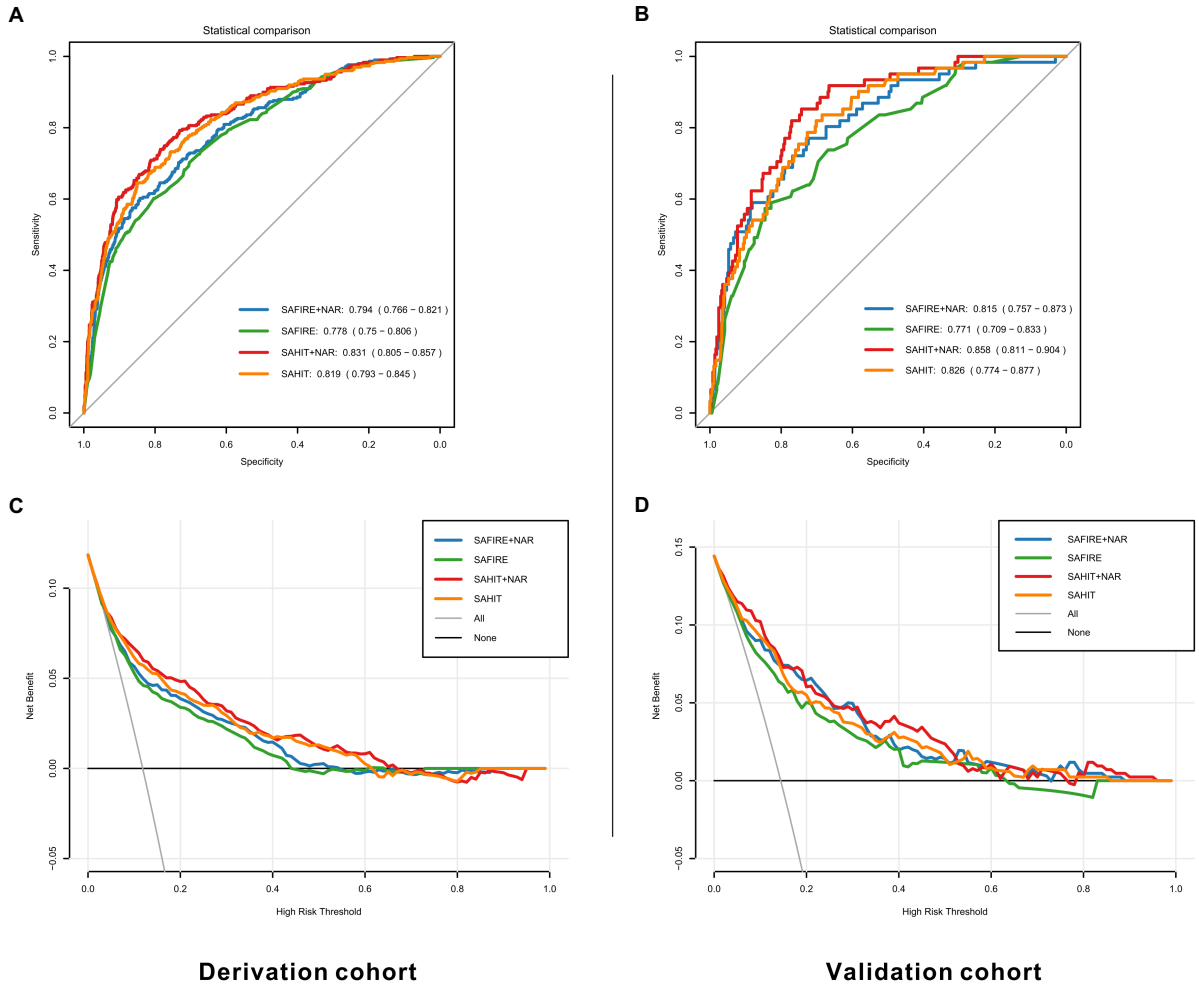
ROC curves and decision curves for original and modified models in the derivation **(A,C)** and validation cohorts **(B,D)**. SAHIT, original Subarachnoid Hemorrhage International Trialists’ model; SAFIRE, the SAFIRE grading scale; NAR, neutrophil-to-albumin ratio.

Compared with the original SAFIRE model, the addition of NAR (NRI = 0.073, *p* = 0.002), MLR (NRI = 0.041, *p* = 0.024), and NLR (NRI = 0.045, *p* = 0.020) could improve the reclassification and the addition of NAR showed the most remarkable reclassification improvement among them. Supplementary Table S6 shows that including NAR as an additional predictor led to 22 (7.36%) extra deaths being classified into a higher risk category, although 2 (0.09%) extra survivors were reclassified into a higher risk category. As shown in Figure 4C, SAFIRE+NAR showed a higher net benefit than the original model.

#### SAHIT + inflammatory markers

As shown in Table 3, the AUC value of the SAHIT model in the derivation cohort (AUC = 0.819, 95% CI 0.793–0.845) was also comparable with that previously reported. The addition of inflammatory markers all improved the modified predictive models. Among the four markers, adding NAR to the SAFIRE and SAHIT models acquired the highest AUC development (SAHIT+NAR vs. SAHIT: ∆AUC = 0.012, *p* = 0.016) and the highest IDI (SAHIT+NAR vs. SAHIT: IDI = 0.023, *p* < 0.001).

**Table 3:** Performance of SAHIT and SAHIT + inflammatory biomarkers models.

Models	Discrimination				Reclassification		Calibration
	AUC (95% CI)	*p* value	IDI (95% CI)	*p* value	Categorical NRI (95% CI)	*p* value	Brier score
*Derivation cohort*							
SAHIT	0.819 (0.793–0.845)	Ref.	Ref.		Ref.		0.083
SAHIT+NAR	0.831 (0.805–0.857)	0.016	0.023 (0.013–0.032)	<0.001	0.0810 (0.038–0.124)	<0.001	0.081
SAHIT+MLR	0.828 (0.802–0.853)	0.015	0.010 (0.002–0.018)	0.012	0.0402 (−0.004–0.084)	0.072	0.082
SAHIT+NLR	0.827 (0.802–0.853)	0.015	0.011 (0.004–0.019)	0.003	0.0554 (0.013–0.098)	0.011	0.082
SAHIT+SII	0.824 (0.798–0.850)	0.103	0.009 (0.003–0.015)	0.004	0.0324 (−0.008–0.073)	0.113	0.082
*Validation cohort*							
SAHIT	0.826 (0.774–0.877)	Ref.	Ref.		Ref.		0.099
SAHIT+NAR	0.858 (0.811–0.904)	0.004	0.053 (0.027–0.079)	<0.001	0.1505 (0.029–0.272)	0.015	0.094
SAHIT+MLR	0.830 (0.778–0.882)	0.597	0.006 (−0.011–0.023)	0.478	0.0519 (−0.048–0.152)	0.308	0.100
SAHIT+NLR	0.829 (0.776–0.881)	0.660	0.018 (−0.001–0.036)	0.056	0.0328 (−0.050–0.115)	0.437	0.098
SAHIT+SII	0.828 (0.775–0.881)	0.719	0.019 (0.002–0.036)	0.031	0.0357 (−0.040–0.112)	0.356	0.098

NAR, neutrophil-to-albumin ratio; MLR, monocyte-to-lymphocyte ratio; NLR, neutrophil-to-lymphocyte ratio; SII, systemic immune-inflammation index; AUC, the area under the receiver operator characteristics curve; IDI, integrated discrimination improvement; NRI, net reclassification improvement; CI, confidence interval.

Compared with the original SAHIT model, only the addition of NAR (NRI = 0.081, *p* < 0.001) and NLR (NRI = 0.055, *p* = 0.011) could improve the reclassification, and the NRI of addition of NAR was still the highest. As shown in Supplementary Table S7, SAHIT+NAR reclassified 24 (1.08%) survivors into a lower risk category and 21 (7.02%) deaths into a higher risk category, which meant both specificity and sensitivity was enhanced. As shown in Figure 4C, including NAR enhanced the net benefit compared with the original SAHIT model, which meant greater clinical usefulness.

### Model validation

#### SAFIRE + inflammatory markers

In the internal validation, for all SAFIRE+NAR models using the derivation cohort data, the mean AUC was 0.785, and the average error was 11.8%. In the temporal validation, the performances of the developed models using the validation cohort data were comparable to those using the derivation cohort data. As shown in Table 2, only the addition of NAR improved the discrimination ability of the SAFIRE model (SAFIRE+NAR vs. SAFIRE: ∆AUC = 0.044, *p* = 0.001; IDI = 0.057, *p* < 0.001). Moreover, including NAR as an additional predictor improved the reclassification ability of the SAFIRE model (NRI = 0.101, *p* = 0.044), and details of the reclassification improvement were shown in Supplementary Table S8. Net benefit was also improved, as shown in Figure 4D.

#### SAHIT + inflammatory markers

In the internal validation, for all SAHIT+NAR models, the mean AUC was 0.820, and the average error was 10.7%. In the temporal validation, the performances of the developed models using the validation cohort data were comparable to those using the derivation cohort data. As shown in Table 3, only the addition of NAR improved the discrimination ability of the SAHIT model (SAHIT+NAR vs. SAHIT: ∆AUC = 0.032, *p* = 0.004; IDI = 0.053, *p* < 0.001). Moreover, including NAR as an additional predictor improved the reclassification ability of the SAHIT model (NRI = 0.151, *p* = 0.015), and details of the reclassification improvement were shown in Supplementary Table S9. According to the DCA in Figure 4D, SAHIT+NAR also showed higher clinical usefulness in the validation cohort.

### Model calibration

We demonstrate the calibration plot in Supplementary Figures S3, S4, and there was no significant evidence of miscalibration. None of the original and developed models got a brier score over 0.25, where lower scores signify better calibration.

### Sensitivity analysis

In the sensitivity analysis, as shown in Supplementary Tables S10, S11, adding markers as categorical variables reduced the improvement of the predictive ability. Meanwhile, the addition of the interaction terms, including NAR and age, WFNS grade, Fisher grade, or treatment, failed to develop the SAFIRE+NAR or SAHIT+NAR model (Supplementary Table S12).

### Model presentation

To better use the developed SAFIRE and SAHIT model, we developed two web-based nomograms, accessible at https://sahit-nar.shinyapps.io/SAHIT-NAR/ and https://sahit-nar.shinyapps.io/SAFIRE-NAR/. The presentation of the webs was presented in Supplementary Figures S5, S6.

## Discussion

This study demonstrated that inflammatory markers could improve the predictive effectiveness of existing models, including discrimination, reclassification, and clinical usefulness. The predictive ability of various inflammatory markers was compared through temporal validation, and it was found that NAR had the best predictive improvement ability. An online calculator was developed for the improved models to facilitate further validation and application.

The involvement of inflammatory response in the acute phase of aSAH may be the source of the ability of inflammatory indicators to predict short-term outcomes of aSAH (23). Studies have reported a peak of inflammatory cytokines within 48 h after the onset of aSAH (24). An excessive inflammatory response leads to a poor prognosis for aSAH (25). Due to the destruction of BBB, peripheral immune cells and their products will also affect the central nervous system. Neutrophils produce oxygen-free radicals and proteolytic enzymes that damage neurons and endothelial cells (26). In recent studies, albumin has been suggested to have a possible protective effect on BBB (27). Many clinical studies have also shown that high neutrophils are associated with prognosis in patients with aSAH (28), while hypoproteinemia is associated with infection during hospitalization (29, 30). This may be the NAR’s mechanism for predicting the outcome of aSAH.

Many previous studies have compared the predictive effects of inflammatory factors alone instead of included in a complete model (31, 32). As shown in Figure 2, Tables 2, 3, we found that the predictive power of inflammatory markers alone was not parallel to their ability to improve the predictive models. In a prediction model proposed by Lai et al. (33), although NLR was included, it was not reported how the inclusion of NLR improved the prediction efficiency of the model. Similarly, in the TAPS model presented by Li et al. (34), the contribution of white blood cells (WBC) to the model was not reported. Moreover, the same predictors (NLR and WBC) presented different predictive performances in the two studies. When demographic information, imaging information, clinical status, laboratory examination, and other indicators of different dimensions are combined, it was unknown whether inflammatory factors could improve the prediction effect of the original model. Therefore, it was necessary to quantify the predictive value of inflammatory factors in the same basic model. Figure 3 showed that in the SAFIRE and SAHIT models, WFNS always had the highest predictive ability. In contrast, the contribution of variables including age, aneurysm location, and size to the model lagged far behind that of WFNS. As shown in Supplementary Figure S2, after adding inflammatory markers to the models and comparing their partial R^2^, we found that the contribution of all inflammatory markers except SII ranked in the top three, and NAR had the highest contribution among them.

At the same time, we compared the differentiation ability of inflammatory factors and, furtherly, their reclassification ability. NRI can reflect the degree to which the improved model differentiates patients at different risk levels. Although NRI has been used in previous studies (35, 36), these studies reported continuous NRI, which is less explanatory than categorical NRI. We found 7–8% NRI for including NAR in the derivation cohort and 10–15% NRI in the validation cohort. Further reclassification analysis found that NAR was better able to identify high-risk (>65%) aSAH patients, which can help to distinguish early and intervene early in clinical practice. In addition, we demonstrated that NAR is a stable and reliable predictor across periods.

Apart from the outstanding discriminative and reclassification ability, NAR also had a significant value in clinical application in other aspects. First, NAR is an inexpensive and easy-to-use inflammatory marker. Neutrophil and albumin levels are routine in-hospital tests for aSAH patients, and the calculation is simple. Moreover, neutrophil and albumin levels are easy targets for clinical intervention. Human albumin treatment can be used to treat hypoalbuminemia. The 1.25 g/kg/day albumin therapy for SAH patients was reported to be tolerable without major complications and might be neuroprotective (37). Furthermore, neutrophil depletion following SAH was suggested to increase memory *via* NMDA receptors (38). However, this biomarker ratio still could not be directly applied in clinical practice. It should be furtherly proven beneficial in the laboratory and then carefully verified in the clinical trials.

This study has numerous strengths. Our study was based on a 10 year large cohort study, supporting our temporal validation. In addition, we obtained the accurate survival status of the enrolled patients at 3 months through the household registration system. Third, we used various methods to quantify how inflammatory factors improved the prediction model. The web-based prognostic calculator can also improve the clinical application value of this study.

However, our study had some limitations. This study was a single-center retrospective study, which could not support us in conducting geographical validation. Further multi-center studies could overcome the limitations of single-center temporal validation. In addition, due to retrospective collection, part of the data was lost. Although multiple imputations were carried out, the feasibility of the research conclusion was reduced. Third, we did not obtain the functional outcome of patients, which prevented us from proving that inflammatory factors were equally good at predicting the functional outcome of aSAH patients.

This study suggested that more attention should be paid to inflammatory indicators when establishing aSAH prediction models. We also preliminarily demonstrated that the combined use of NAR improved the predictive performance of existing prediction models. NAR was an inexpensive and convenient early laboratory index that deserves further clinical validation in more prospective multicenter studies.

## Conclusion

This study illustrated that among the inflammatory markers associated with aSAH prognosis, NAR could improve the SAFIRE and SAHIT models for the 3-month mortality of aSAH.

## Data availability statement

The original contributions presented in the study are included in the article/Supplementary material, further inquiries can be directed to the corresponding author.

## Ethics statement

The studies involving human participants were reviewed and approved by the ethics committee of West China Hospital (No. 20211701). Written informed consent for participation was not required for this study in accordance with the national legislation and the institutional requirements.

## Author contributions

RZ, YZ, CY, LM, and FF: study concept and design. RZ, YZ, ZL, YP, YH, and JY: acquisition, analysis, or interpretation of data. RZ, YZ, and ZL: statistical analysis. RZ: drafting of the manuscript. RZ, YZ, ZL, YP, YH, JY, CY, LM, and FF: critical revision of the manuscript for important intellectual content. All authors contributed to the article and approved the submitted version.

## Funding

This work is supported by National Key R&D Program of China (2018YFA0108604) (LM), National Natural Science Foundation of China (82271364) (YZ), the innovation team project of Affiliated Hospital of Clinical Medicine College of Chengdu University (CDFYCX202202) (YZ), the project of Sichuan Science and Technology Bureau (22ZDYF0798) (FF), and Clinical Incubation Program of West China Hospital, SCU (2018HXFU008) (LM).

## Conflict of interest

The authors declare that the research was conducted in the absence of any commercial or financial relationships that could be construed as a potential conflict of interest.

## Publisher’s note

All claims expressed in this article are solely those of the authors and do not necessarily represent those of their affiliated organizations, or those of the publisher, the editors and the reviewers. Any product that may be evaluated in this article, or claim that may be made by its manufacturer, is not guaranteed or endorsed by the publisher.

## Abbreviations

aSAH: aneurysmal subarachnoid hemorrhage
NAR: neutrophil-to-albumin ratio
SII: systemic immune-inflammation index
WFNS: World Federation of Neurological Surgeons
AUC: area under the receiver operator characteristics curve
IDI: integrated discrimination improvement
NRI: net reclassification improvement
DCA: decision-curve analysis
VIF: variance inflation factor
