# Supplementary material for: Improving the models for prognosis of aneurysmal subarachnoid hemorrhage with the neutrophil-to-albumin ratio
Source: Front Neurol. 2023 Mar 24;14:1078926. doi: 10.3389/fneur.2023.1078926 (PMC10079994; doi:10.3389/fneur.2023.1078926)
Supplement: Supplementary file 2 [file Data_Sheet_2.PDF]

SUPPLEMENTAL MATERIAL

Appendix

Table S1 Discriminative capacity of inflammatory biomarkers

Table S2 Collinearity test results of original SAFIRE predictors and biomarkers

Table S3 Collinearity test results of original SAHIT predictors and biomarkers

Table S4 Details on SAFIRE and SAFIRE + NAR models

Table S5 Details on SAHIT and SAHIT + NAR models

Table S6 Net Reclassification Improvement for SAFIRE + NAR model

Table S7 Net Reclassification Improvement for SAHIT + NAR model

Table S8 Net Reclassification Improvement for SAFIRE + NAR model in the validation cohort

Table S9 Net Reclassification Improvement for SAHIT + NAR model in the validation cohort

Table S10 Performance of SAFIRE and SAFIRE + inflammatory biomarkers models (biomarkers included as categorical variables)

Table S11 Performance of SAHIT and SAHIT + inflammatory biomarkers models (biomarkers included as categorical variables)

Table S12 Results of test of potentially meaningful interaction effects after addition of NAR

Figure S1 Calculation methods for the different combinations of inflammatory markers

Figure S2 General dominance of predictors reported as partial R2 statistic after addition of MLR, NLR or SII

Figure S3 Calibration plots of original and modified SAFIRE models in the derivation cohort and validation cohorts

Figure S4 Calibration plots of original and modified SAHIT models in the derivation cohort and validation cohorts

Figure S5 Web of the modified SAFIRE model

Figure S6 Web of the modified SAHIT model

Table S1 Discriminative capacity of inflammatory biomarkers

Biomarkers	AUC (95% CI)	Youden Index	Threshold	Specificity	Sensitivity
Derivation cohort					
NAR	0.707 (0.673 - 0.74)	0.334	0.252	0.712	0.622
SIRI	0.696 (0.663 - 0.73)	0.305	7.004	0.804	0.502
NLR	0.687 (0.654 - 0.72)	0.286	9.028	0.637	0.649
SII	0.656 (0.622 - 0.69)	0.245	1.986	0.764	0.482
MLR	0.656 (0.621 - 0.692)	0.253	0.674	0.805	0.448
PLR	0.562 (0.525 - 0.599)	0.123	242.258	0.829	0.294
Validation cohort					
NAR	0.77 (0.712 - 0.828)	0.438	0.278	0.812	0.627
SIRI	0.742 (0.688 - 0.797)	0.346	8.565	0.839	0.507
NLR	0.701 (0.64 - 0.762)	0.298	10.696	0.658	0.640
SII	0.683 (0.622 - 0.744)	0.287	1.455	0.567	0.720
MLR	0.655 (0.589 - 0.721)	0.269	0.658	0.749	0.520
PLR	0.453 (0.381 - 0.524)	0.034	163.057	0.501	0.533

NAR, neutrophil-to-albumin ratio; MLR, monocyte-to-lymphocyte ratio; NLR, neutrophil-to-lymphocyte ratio; SII, systemic immune-inflammation index; PLR, platelet-to-lymphocyte ratio; AUC, area under the receiver operator characteristics curve; CI, confidence interval.

Table S2 Collinearity test results of original SAFIRE predictors and biomarkers

Variables	VIF			
	SAFIRE+NAR	SAFIRE+MLR	SAFIRE+NLR	SAFIRE+SII
Original SAFIRE predictors				
Age 50-60 y	1.252	1.250	1.249	1.251
Age 60-70 y	1.259	1.257	1.257	1.260
Age ⩾70 y	1.164	1.162	1.162	1.167
Size 10-20 mm	1.014	1.015	1.017	1.015
Size ⩾20 mm	1.018	1.015	1.015	1.017
Fisher grade Ⅳ	1.145	1.133	1.144	1.133
WFNS grade Ⅱ	1.816	1.811	1.812	1.812
WFNS grade Ⅲ	1.187	1.173	1.178	1.175
WFNS grade Ⅳ	1.990	1.984	1.982	1.981
WFNS grade Ⅴ	1.460	1.381	1.391	1.365
Additional biomarkers				
NAR	1.285	—	—	—
MLR	—	1.140	—	—
NLR	—	—	1.193	—
SII	—	—	—	1.137

VIF, variance inflation factor; WFNS, World Federation of Neurological Surgeons; NAR, neutrophil-to-albumin ratio; MLR, monocyte-to-lymphocyte ratio; NLR, neutrophil-to-lymphocyte ratio; SII, systemic immune-inflammation index; PLR, platelet-to-lymphocyte ratio.

Table S3 Collinearity test results of original SAHIT predictors and biomarkers

Variables	VIF			
	SAHIT+NAR	SAHIT+MLR	SAHIT+NLR	SAHIT+SII
Original SAHIT predictors				
Age	1.076	1.073	1.073	1.079
Hypertension Yes	1.039	1.037	1.037	1.037
Location Posterior circulation	1.073	1.074	1.073	1.074
Size 13-24 mm	1.013	1.013	1.013	1.013
Size ≥24 mm	1.013	1.012	1.012	1.012
Fisher grade Ⅱ	1.026	1.022	1.024	1.022
Fisher grade Ⅲ	1.493	1.493	1.493	1.494
Fisher grade Ⅳ	1.619	1.598	1.614	1.597
WFNS grade Ⅱ	1.830	1.827	1.828	1.828
WFNS grade Ⅲ	1.188	1.175	1.179	1.176
WFNS grade Ⅳ	2.031	2.026	2.024	2.023
WFNS grade Ⅴ	1.500	1.417	1.430	1.403
Operation Clip	1.735	1.736	1.736	1.738
Operation Coil	1.689	1.691	1.693	1.694
Additional biomarkers				
NAR	1.307	—	—	—
MLR	—	1.155	—	—
NLR	—	—	1.210	—
SII	—	—	—	1.147

VIF, variance inflation factor; WFNS, World Federation of Neurological Surgeons; NAR, neutrophil-to-albumin ratio; MLR, monocyte-to-lymphocyte ratio; NLR, neutrophil-to-lymphocyte ratio; SII, systemic immune-inflammation index; PLR, platelet-to-lymphocyte ratio.

Table S4 Details on SAFIRE and SAFIRE + NAR models

Characteristic	SAFIRE			SAFIRE + NAR		
	β coefficient	OR	95% CI	β coefficient	OR	95% CI
Intercept	-3.01	—	—	-4.04	—	—
Age ≤50 y	Ref.					
Age 50-60 y	0.17	1.19	0.83, 1.69	0.29	1.33	0.93, 1.91
Age 60-70 y	0.35	1.42	1.00, 2.00	0.44	1.55	1.09, 2.21
Age ≥70 y	0.90	2.46	1.65, 3.65	1.04	2.82	1.88, 4.21
Size <10 mm	Ref.					
Size 10-20 mm	0.32	1.38	0.97, 1.93	0.34	1.40	0.98, 1.98
Size ≥20 mm	0.70	2.01	1.21, 3.24	0.80	2.23	1.34, 3.62
Fisher grade Ⅰ-Ⅲ	Ref.					
Fisher grade Ⅳ	0.74	2.09	1.54, 2.85	0.65	1.92	1.41, 2.64
WFNS grade Ⅰ	Ref.					
WFNS grade Ⅱ	-0.98	0.38	0.10, 0.90	-0.89	0.41	0.11, 0.99
WFNS grade Ⅲ	0.55	1.73	1.26, 2.39	0.44	1.56	1.13, 2.15
WFNS grade Ⅳ	1.36	3.89	2.00, 10.4	1.30	3.68	1.88, 9.89
WFNS grade Ⅴ	1.39	4.03	3.11, 5.22	1.06	2.89	2.19, 3.81
NAR	—	—	—	3.78	43.60	14.1, 136

WFNS, World Federation of Neurological Surgeons; NAR, neutrophil-to-albumin ratio; MLR, monocyte-to-lymphocyte ratio; NLR, neutrophil-to-lymphocyte ratio; SII, systemic immune-inflammation index; PLR, platelet-to-lymphocyte ratio; ; OR, odds ratio; CI, confidence interval.

Table S5 Details on SAHIT and SAHIT + NAR models

Characteristic	SAHIT			SAHIT + NAR		
	β coefficient	OR	95% CI	β coefficient	OR	95% CI
Intercept	-2.48	—	—	-3.58	—	—
Age	0.01	1.01	1.00, 1.03	0.02	1.02	1.01, 1.03
Hypertension No	Ref.					
Hypertension Yes	-0.16	0.85	0.62, 1.16	-0.21	0.81	0.59, 1.10
Location Anterior circulation	Ref.					
Location Posterior circulation	0.10	1.10	0.76, 1.58	0.10	1.10	0.75, 1.59
Size ≤13 mm	Ref.					
Size 13-24 mm	0.80	2.24	1.44, 3.41	0.85	2.34	1.49, 3.61
Size ≥24 mm	0.99	2.69	1.57, 4.48	1.08	2.94	1.71, 4.95
Fisher grade Ⅰ	Ref.					
Fisher grade Ⅱ	0.21	1.24	0.81, 1.92	0.14	1.16	0.75, 1.81
Fisher grade Ⅲ	0.18	1.19	0.69, 1.94	0.23	1.26	0.73, 2.06
Fisher grade Ⅳ	0.98	2.67	1.55, 5.17	0.80	2.23	1.30, 4.34
WFNS grade Ⅰ	Ref.					
WFNS grade Ⅱ	-0.89	0.41	0.11, 1.00	-0.83	0.43	0.11, 1.07
WFNS grade Ⅲ	0.60	1.83	1.31, 2.54	0.49	1.63	1.17, 2.29
WFNS grade Ⅳ	1.10	3.01	1.53, 8.12	1.08	2.93	1.47, 7.98
WFNS grade Ⅴ	1.18	3.26	2.49, 4.27	0.84	2.33	1.74, 3.11
Operation No treatment	Ref.					
Operation Clip	-1.42	0.24	0.18, 0.33	-1.43	0.24	0.17, 0.33
Operation Coil	-1.07	0.34	0.21, 0.54	-1.09	0.34	0.21, 0.53
NAR	—	—	—	3.74	42.00	12.9, 137

WFNS, World Federation of Neurological Surgeons; NAR, neutrophil-to-albumin ratio; MLR, monocyte-to-lymphocyte ratio; NLR, neutrophil-to-lymphocyte ratio; SII, systemic immune-inflammation index; PLR, platelet-to-lymphocyte ratio; ; OR, odds ratio; CI, confidence interval.

Table S6 Net Reclassification Improvement for SAFIRE + NAR model in the derivation cohort

Survival within 3 months	SAFIRE + NAR				
SAFIRE	Risk of 3-months mortality	<10%	10%–65%	>65%	Reclassified rate, %
	<10%	1479	113	0	7
	10%–65%	117	507	7	20
	>65%	0	1	2	33
Death within 3 months	SAFIRE + NAR				
SAFIRE	Risk of 3-months mortality	<10%	10%–65%	>65%	Reclassified rate, %
	<10%	73	22	0	23
	10%–65%	13	177	13	13
	>65%	0	0	1	0
NRI (95% CI)	0.073 (0.026-0.119) P=0.002				

NAR, neutrophil-to-albumin ratio; NRI, net reclassification improvement; CI, confidence interval.

Table S7 Net Reclassification Improvement for SAHIT + NAR model in the derivation cohort

Survival within 3 months	SAHIT + NAR				
SAHIT	Risk of 3-months mortality	<10%	10%–65%	>65%	Reclassified rate, %
	<10%	1543	91	0	6
	10%–65%	117	449	10	22
	>65%	0	8	8	50
Death within 3 months	SAHIT + NAR				
SAHIT	Risk of 3-months mortality	<10%	10%–65%	>65%	Reclassified rate, %
	<10%	62	16	0	21
	10%–65%	6	175	15	11
	>65%	0	4	21	16
NRI (95% CI)	0.081 (0.038-0.124) P<0.001				

NAR, neutrophil-to-albumin ratio; NRI, net reclassification improvement; CI, confidence interval.

Table S8 Net Reclassification Improvement for SAFIRE + NAR model in the validation cohort

Survival within 3 months	SAFIRE + NAR				
SAFIRE	Risk of 3-months mortality	<10%	10%–65%	>65%	Reclassified rate, %
	<10%	219	20	0	8
	10%–65%	17	103	2	16
	>65%	0	0	1	0
**Death within 3 months**	**SAFIRE + NAR**				
SAFIRE	Risk of 3-months mortality	<10%	10%–65%	>65%	Reclassified rate, %
	<10%	11	5	0	31
	10%–65%	1	41	3	9
	>65%	0	0	0	0
NRI (95% CI)	0.1009 (0.0029-0.1989) P=0.04				

NAR, neutrophil-to-albumin ratio; NRI, net reclassification improvement; CI, confidence interval.

Table S9 Net Reclassification Improvement for SAHIT + NAR model in the validation cohort

Survival within 3 months	SAHIT + NAR				
SAHIT	Risk of 3-months mortality	<10%	10%–65%	>65%	Reclassified rate, %
	<10%	210	17	0	7
	10%–65%	26	100	3	22
	>65%	0	1	5	17
Death within 3 months	SAHIT + NAR				
SAHIT	Risk of 3-months mortality	<10%	10%–65%	>65%	Reclassified rate, %
	<10%	5	4	0	44
	10%–65%	0	36	7	16
	>65%	0	3	6	33
NRI (95% CI)	0.1505 (0.0291-0.2719) P=0.015				

NAR, neutrophil-to-albumin ratio; NRI, net reclassification improvement; CI, confidence interval.

Table S10 Performance of SAFIRE and SAFIRE + inflammatory biomarkers models (biomarkers included as categorical variables)

Models	Discrimination				Reclassification		Calibration
	AUC (95% CI)	*P* value	IDI (95% CI)	*P* value	Categorical NRI (95% CI)	*P* value	Brier score
Derivation cohort							
SAFIRE	0.778 (0.750-0.806)	Ref.	Ref.		Ref.		0.090
SAFIRE+NAR	0.787 (0.758-0.815)	0.159	0.0174 (0.0115-0.0233)	<0.001	0.034 (-0.0124-0.0804)	0.151	0.088
SAFIRE+MLR	0.789 (0.762-0.816)	0.028	0.0099 (0.0033-0.0165)	0.003	0.0146 (-0.0205-0.0497)	0.414	0.089
SAFIRE+NLR	0.787 (0.760-0.814)	0.084	0.0066 (0.0021-0.0111)	0.004	0.0081 (-0.0320-0.0482)	0.693	0.089
SAFIRE+SII	0.784 (0.756-0.811)	0.305	0.0161 (0.0094-0.0227)	<0.001	0.0187 (-0.0248-0.0622)	0.400	0.088
Validation cohort							
SAFIRE	0.771 (0.709-0.833)	Ref.	Ref.		Ref.		0.108
SAFIRE+NAR	0.805 (0.746-0.865)	0.008	0.0273 (0.0147-0.0400)	<0.001	0.0514 (-0.0343-0.1371)	0.240	0.103
SAFIRE+MLR	0.791 (0.732-0.850)	0.098	0.0082 (-0.0082-0.0245)	0.329	-0.0387 (-0.1106-0.0332)	0.292	0.107
SAFIRE+NLR	0.777 (0.716-0.838)	0.595	0.0054 (-0.0049-0.0157)	0.301	-0.0499 (-0.1441-0.0442)	0.299	0.108
SAFIRE+SII	0.781 (0.721-0.842)	0.451	0.0156 (0-0.0312)	0.050	0.0024 (-0.1060-0.1108)	0.965	0.108

NAR, neutrophil-to-albumin ratio; MLR, monocyte-to-lymphocyte ratio; NLR, neutrophil-to-lymphocyte ratio; SII, systemic immune-inflammation index; AUC, area under the receiver operator characteristics curve; IDI, integrated discrimination improvement; NRI, net reclassification improvement; CI, confidence interval.

Table S11 Performance of SAHIT and SAHIT + inflammatory biomarkers models (biomarkers included as categorical variables)

Models	Discrimination				Reclassification		Calibration
	AUC (95% CI)	*P* value	IDI (95% CI)	*P* value	Categorical NRI (95% CI)	*P* value	Brier score
Derivation cohort							
SAHIT	0.819 (0.793-0.845)	Ref.	Ref.		Ref.		0.083
SAHIT+NAR	0.825 (0.799-0.851)	0.212	0.0187 (0.0122-0.0252)	<0.001	0.0784 (0.0306-0.1262)	0.001	0.081
SAHIT+MLR	0.826 (0.800-0.851)	0.035	0.0058 (0.0004-0.0112)	0.035	0.0447 (-0.0021-0.0914)	0.061	0.083
SAHIT+NLR	0.825 (0.800-0.851)	0.087	0.0068 (0.0020-0.0116)	0.005	0.0245 (-0.0136-0.0627)	0.208	0.082
SAHIT+SII	0.821 (0.795-0.848)	0.483	0.0113 (0.0055-0.0171)	<0.001	0.0502 (0.0037-0.0966)	0.034	0.082
Validation cohort							
SAHIT	0.826 (0.774-0.877)	Ref.	Ref.		Ref.		0.099
SAHIT+NAR	0.848 (0.799-0.897)	0.02	0.0308 (0.0153-0.0463)	<0.001	0.0820 (-0.0310-0.1949)	0.155	0.095
SAHIT+MLR	0.829 (0.776-0.881)	0.661	0.0083 (-0.0058-0.0225)	0.249	0.0138 (-0.0825-0.1102)	0.779	0.098
SAHIT+NLR	0.828 (0.777-0.879)	0.756	0.0075 (-0.0044-0.0194)	0.215	0.0079 (-0.0646-0.0804)	0.830	0.099
SAHIT+SII	0.823 (0.768-0.878)	0.742	0.0151 (0.0007-0.0294)	0.039	0.0053 (-0.0974-0.1081)	0.919	0.098

NAR, neutrophil-to-albumin ratio; MLR, monocyte-to-lymphocyte ratio; NLR, neutrophil-to-lymphocyte ratio; SII, systemic immune-inflammation index; AUC, area under the receiver operator characteristics curve; IDI, integrated discrimination improvement; NRI, net reclassification improvement; CI, confidence interval.

Table S12 Results of test of potentially meaningful interaction effects after addition of NAR

Models	AUC (95% CI)	*P* value
SAFIRE+NAR	0.794 (0.766-0.821)	Ref.
SAFIRE+NAR+NAR & age	0.793 (0.765-0.820)	0.277
SAFIRE+NAR+NAR & WFNS grade	0.796 (0.768-0.823)	0.166
SAFIRE+NAR+NAR & Fisher grade	0.793 (0.765-0.821)	0.227
SAHIT+NAR	0.831 (0.805-0.857)	Ref.
SAHIT+NAR+NAR & age	0.831 (0.805-0.857)	0.823
SAHIT+NAR+NAR & WFNS grade	0.832 (0.806-0.857)	0.529
SAHIT+NAR+NAR & Fisher grade	0.832 (0.807-0.858)	0.401
SAHIT+NAR+NAR & operation	0.834 (0.809-0.860)	0.273

NAR, neutrophil-to-albumin ratio; WFNS, World Federation of Neurological Surgeons; AUC, area under the receiver operator characteristics curve; CI, confidence interval.

NAR	=	Neutrophil (10^9/L)	/	Albumin (g/L)				
NLR	=	Neutrophil (10^9/L)	/	Lymphocyte (10^9/L)				
PLR	=	Platelet (10^9/L)	/	Lymphocyte (10^9/L)				
MLR	=	Monocyte (10^9/L)	/	Lymphocyte (10^9/L)				
SII	=	Platelet (10^9/L)	x	Neutrophil (10^9/L)	/	Lymphocyte (10^9/L)	/	1000
SIRI	=	Neutrophil (10^9/L)	x	Monocyte (10^9/L)	/	Lymphocyte (10^9/L)		

Figure S1 Calculation methods for the different combinations of inflammatory biomarkers

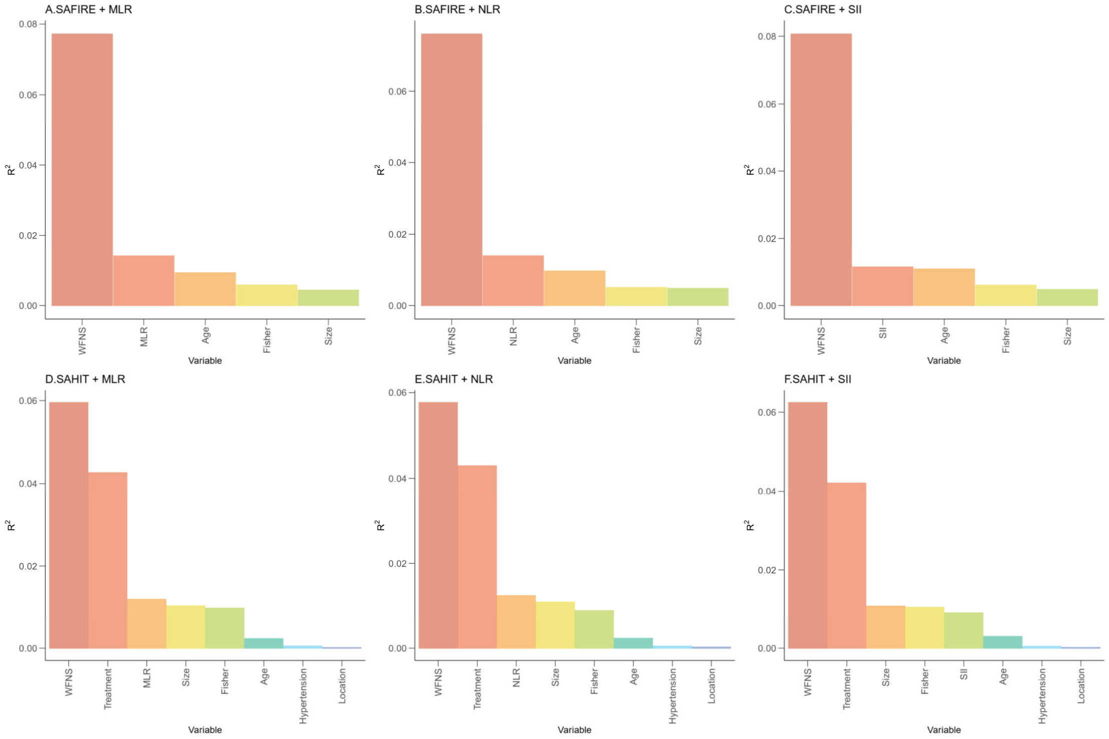

Figure S2 General dominance of predictors reported as partial R2 statistic after addition of MLR, NLR or SII

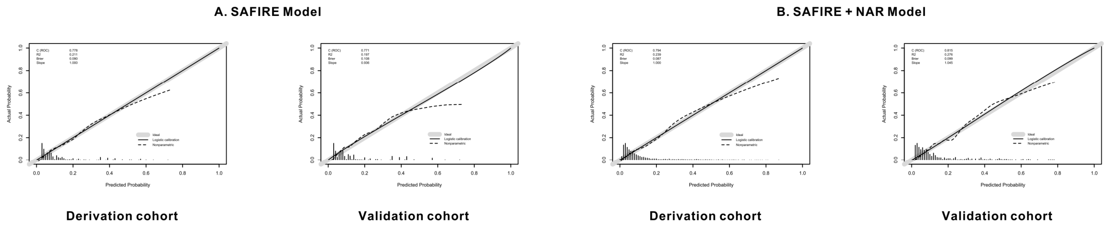

Figure S3 Calibration plots of original and modified SAFIRE models in the derivation cohort and validation cohorts

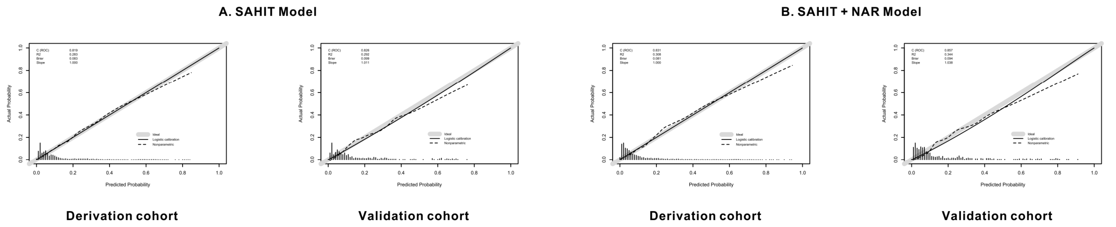

Figure S4 Calibration plots of original and modified SAHIT models in the derivation cohort and validation cohorts

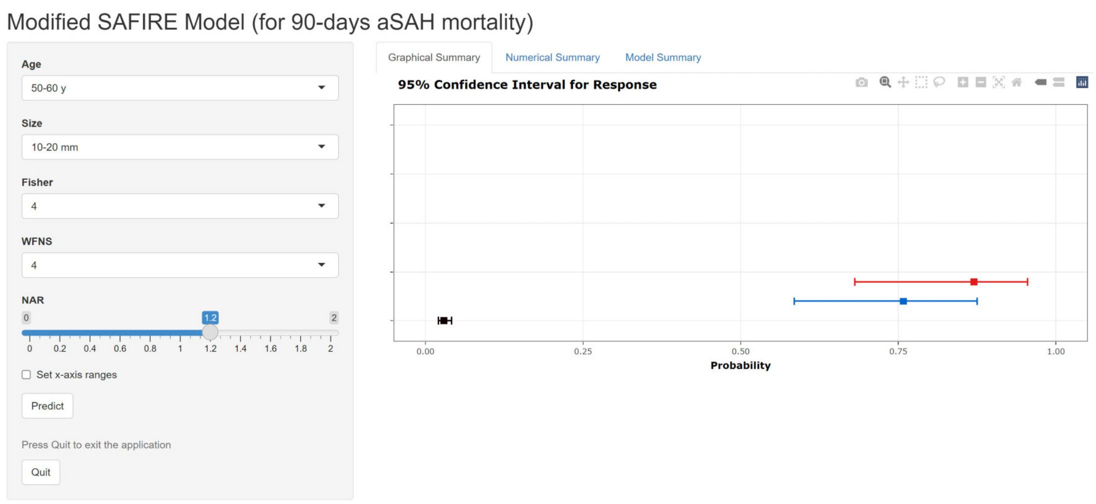

Figure S5 Web of the modified SAFIRE model

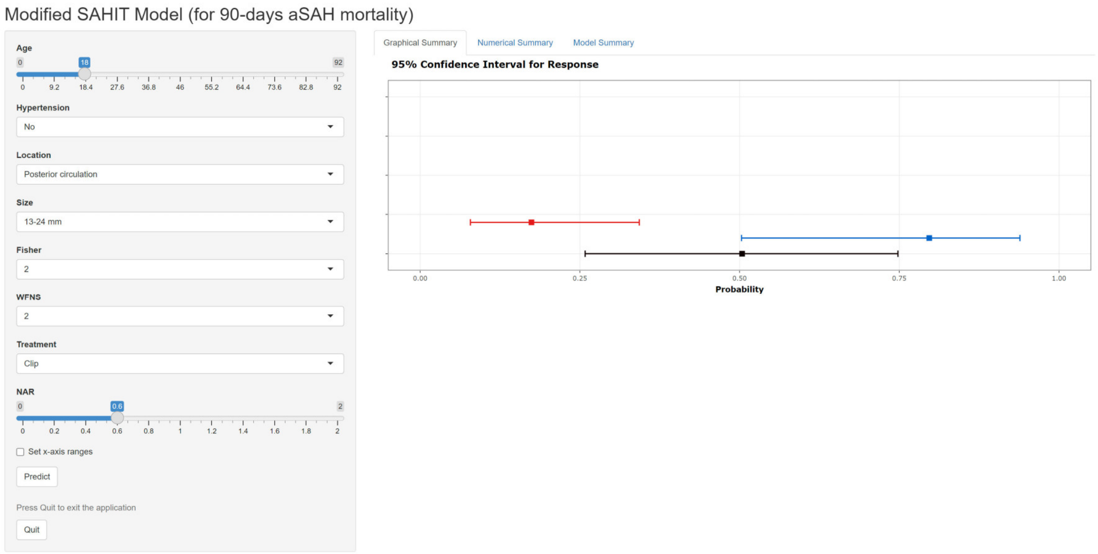

Figure S6 Web of the modified SAHIT model
